# Criminal, Legal, and Ethical Kidney Donation and Transplantation: A Conceptual Framework to Enable Innovation

**DOI:** 10.3389/ti.2022.10551

**Published:** 2022-06-24

**Authors:** Alvin E. Roth, Ignazio R. Marino, Kimberly D. Krawiec, Michael A. Rees

**Affiliations:** ^1^ Department of Economics, Stanford University, Stanford, CA, United States; ^2^ Department of Surgery, Thomas Jefferson University, Philadelphia, PA, United States; ^3^ School of Law, University of Virginia, Charlottesville, VA, United States; ^4^ Department of Urology, University of Toledo Medical Center, Toledo, OH, United States; ^5^ Alliance for Paired Kidney Donation, Toledo, OH, United States

**Keywords:** innovated-projects, kidney transplantation, ethics, living donor, legal aspects

Criminal, legal, and ethical actions are three very different issues: this applies to all human activities, including living kidney donation and transplantation.

Criminal live donor kidney transplantation happens in countries with illegal black markets for organ transplantation. In these countries, surgeons perform the procedure outside regular medical centers, where donors and recipients receive poor surgical care and no postoperative care. Therefore, patients return to traditional medical institutions with no documentation and often with severe and life-threatening opportunistic infections (1). So far, longstanding efforts to eliminate these markets have failed, despite widespread repugnance to them, and the passage of laws criminalizing payments to donors (2).

Moeindarbari and Feizi (3) discuss the kidney market in Mashad. In Iran, it is legal to pay kidney donors. Transplants and nephrectomies are conducted in well-qualified transplant centers, which are also responsible for postoperative care of donors and recipients. The vast majority of the world transplant community opposes payments to organ donors, whether legal or illegal. Iranians emphasize the difference between criminal and legal live donor kidney transplantation. Many members of the international transplant community have witnessed that the legal live donor kidney transplantation in Iran is conducted with the highest medical and surgical standards.

We strongly believe that international efforts should concentrate on increasing the availability of ethical high-quality live donor kidney transplantation options in all countries.

This is not the same as accepting legalized organ markets, as in Iran. But the present state of the discussion, and its legitimate concern with black markets, has become so dysfunctional that caught in the crossfire of these counterproductive discussions have been other ways of increasing the availability of legal, ethical and safe transplantation and donation. Vigorously opposing criminal black markets should not be conflated with opposing all innovations in living kidney donation that draw closer to the line of valuable consideration. Many recent innovations, such as various forms of kidney exchange, remain inappropriately associated with illegal black markets, when in fact they are opportunities to reduce the demand for illegal black markets.

Kidney exchange has become well established as a standard form of ethical live donor kidney transplantation in several countries, and has led to tens of thousands of additional living donor kidney transplants over the last 2 decades. Yet it is still far from being as available as it could and should be (4). In some countries, non-directed donor chains are not allowed (but these account for the majority of kidney exchange transplants in the United States) (5, 6). Initiating such chains with deceased donor kidneys would further expand their scope (7, 8). In India, the range of family members eligible to be the donor in an incompatible patient-donor pair is more restrictive than those authorized to give a transplant directly: e.g., a patient is authorized to receive a kidney from her uncle if he is a compatible donor, but not to enter into kidney exchange with him if he is incompatible. And many countries do not yet have kidney exchange, such as Brazil and Germany, where kidney exchange remains illegal except in exceptional cases. Many other countries, like Switzerland or Denmark, are too small to be able to offer enough matching opportunities for kidney exchange in a self-sufficient environment. Even the United States is too small to have ready exchange opportunities for the most highly sensitized patients. A limited number of successful international exchanges have taken place, overcoming significant obstacles. For example, a first exchange between Israel and the UAE took place last summer (9). In each case, expanding the opportunity for ethical live donor kidney exchange might give someone a safe, legal and ethical kidney transplant. Any obstacle to ethical kidney transplantation activity supports criminals because it creates demand for an illegal, unsafe, and unethical black market transplant.

The 2017 Statement of the Pontifical Academy of Sciences Summit on Organ Trafficking and Transplant Tourism states that “organ trafficking and human trafficking for the purpose of organ removal” are “crimes against humanity” and specifies what should be considered as qualifying for that designation by recommending: “That all nations and all cultures recognize human trafficking for the purpose of organ removal and organ trafficking, which include the use of organs from executed prisoners and payments to donors or the next of kin of deceased donors, as crimes that should be condemned worldwide and legally prosecuted at the national and international level.”

Note that “crimes against humanity” entered the legal lexicon in the post-World War II Nuremberg trials of Nazi war criminals. There probably is little controversy about extending that term to murdering prisoners for their organs. But is that equivalent to “payments to donors or the next of kin of deceased donors?” Suppose that one of us is called to judge the Nazi war criminals responsible for the Shoah, the Chinese government formerly tolerating the retrieval of organs from executed prisoners (10), and the Iranian government allowing payments to donors, or payments to the next of kin of deceased donors. Are the Nazi, Chinese, and Iranian governments committing the same crimes against humanity?

These types of overbroad generalizations are unhelpful and we agree with the view taken by the American Society of Transplant Surgeons and the American Society of Transplantation calling for exploration of ways to increase legal and ethical transplantation that could involve an “‘Arc of Change’ from removing disincentives to testing incentives.” While not supporting direct payments to donors they write:

*“We believe it important not to conflate the illegal market for organs, which we reject in the strongest possible terms, with the potential in the United States for concerted action to remove all remaining financial disincentives for donors and critically consider testing the impact and acceptability of incentives to increase organ availability in the United States*” (11).

Discussions of black markets are often conducted with reference to the 2008 Declaration of Istanbul, which defines:

“*Organ trafficking is the recruitment, transport, transfer, harboring or receipt of living or deceased persons or their organs by means of the threat or use of force or other forms of coercion, of abduction, of fraud, of deception, of the abuse of power or of a position of vulnerability, or of the giving to, or the receiving by, a third party of payments or benefits to achieve the transfer of control over the potential donor, for the purpose of exploitation by the removal of organs for transplantation*”

and

“*Transplant commercialism is a policy or practice in which an organ is treated as a commodity, including by being bought or sold or used for material gain*” (12).

Moeindarbari and Feizi write that the Iranian market is “In line with the Declaration of Istanbul” because:

“*It authorizes monetary compensation for kidney transplantation but does not tolerate transplant commercialism. Commercialism refers to the possibility within the free-market system to abuse vulnerable people to make a private profit. However, donors in the Iranian Model of Kidney Transplantation are not exploited, but they are supported by law and protected by medical insurance. Therefore, the Iranian Model of Kidney Transplantation adheres to the Declaration of Istanbul*.”

We doubt that all the drafters of the Declaration’s language would agree with this interpretation. But we welcome and encourage more attention to the dangers of black markets and the ways in which increasing safe, legal and ethical transplant opportunities around the world can put an end to criminal black markets, which remain busy and profitable due to the shortage of legal and ethical alternatives.
